# Bonsai: an event-based framework for processing and controlling data streams

**DOI:** 10.3389/fninf.2015.00007

**Published:** 2015-04-08

**Authors:** Gonçalo Lopes, Niccolò Bonacchi, João Frazão, Joana P. Neto, Bassam V. Atallah, Sofia Soares, Luís Moreira, Sara Matias, Pavel M. Itskov, Patrícia A. Correia, Roberto E. Medina, Lorenza Calcaterra, Elena Dreosti, Joseph J. Paton, Adam R. Kampff

**Affiliations:** ^1^Champalimaud Neuroscience Programme, Champalimaud Centre for the UnknownLisbon, Portugal; ^2^Departamento de Ciência dos Materiais, CENIMAT/I3N and CEMOP/UninovaLisbon, Portugal; ^3^Department of Cell and Developmental Biology, University College LondonLondon, UK

**Keywords:** rapid prototyping, data acquisition system, data stream processing, parallel processing, open-source, video tracking, electrophysiology, behavior control

## Abstract

The design of modern scientific experiments requires the control and monitoring of many different data streams. However, the serial execution of programming instructions in a computer makes it a challenge to develop software that can deal with the asynchronous, parallel nature of scientific data. Here we present Bonsai, a modular, high-performance, open-source visual programming framework for the acquisition and online processing of data streams. We describe Bonsai's core principles and architecture and demonstrate how it allows for the rapid and flexible prototyping of integrated experimental designs in neuroscience. We specifically highlight some applications that require the combination of many different hardware and software components, including video tracking of behavior, electrophysiology and closed-loop control of stimulation.

## Introduction

Modern scientific experiments crucially depend on the control and monitoring of many parallel streams of data. Multiple measurement devices, from video cameras, microphones, and pressure sensors to neural electrodes, must simultaneously send their data in real-time to a recording system. General purpose digital computers have gradually replaced many of the specialized analog and digital technologies used for this kind of data acquisition and experiment control, largely due to the flexibility of programming and the exponential growth in computing power. However, the serial nature of programming instructions and shared memory makes it a challenge, even for experienced programmers, to develop software that can elegantly deal with the asynchronous, parallel nature of scientific data.

Another challenge arises from the need for software integration. Each hardware vendor provides their own set of drivers and programming interfaces for configuring and acquiring data from their devices. In addition, the growth of the open-source movement has greatly increased the number of freely available technologies for different data processing domains. Integration of these diverse software and hardware components remains a major challenge for researchers.

These difficulties lead to increased development times when setting up an experiment. Moreover, it requires experimenters to pursue specialized training outside their domain of research. This limits the ability to rapidly prototype and try out new designs and can quickly become the factor limiting the kinds of questions that are amenable to scientific investigation.

Here we describe Bonsai, an open-source visual programming framework for processing data streams (Box 1). The main goal of Bonsai is to simplify and accelerate the development of software for acquiring and processing the many heterogeneous data sources commonly used in (neuro) scientific research. We aim to facilitate the fast implementation of state-of-the-art experimental designs and to encourage the exploration of new paradigms. The framework has already been successfully used for many applications. In the following we will specifically highlight Bonsai's utility in neuroscience for monitoring and controlling a diverse range of behavior and physiology experiments.

Box 1 **Getting Started with Bonsai**

**Community**

The Bonsai framework can be downloaded at https://bitbucket.org/horizongir/bonsai and installed on Windows operating systems starting with Windows 7 and above. The website is organized into different sections: *Downloads* (where the latest installer is located), *Wiki* (with a “Getting Started” guide, tutorials and (FAQ) frequently asked questions), and *Issues* (where bugs can be reported). We have also created a user forum (address is listed in the FAQ section) where the community of Bonsai users have been sharing their feedback, questions and experiences.

A video tutorial introduction to Bonsai is included with this publication (Supplementary Video 1).

**Extending Bonsai**

Bonsai was designed from the outset to support many different layers of extensibility:

- Dataflows: The first layer is through the creation of Bonsai dataflow files themselves. Existing dataflows can be directly reused inside other dataflows as nested nodes. This allows for the sharing of reusable dataflow design patterns between applications.
- Python Scripting: Bonsai supports embedded scripting using IronPython 2.7. Specifically, Bonsai includes three types of Python nodes: *PythonTransform*, *PythonCondition*, and *PythonSink*, which all operate by calling a user-defined Python function described by a script. Below we include a simple example of a *PythonTransform* for rescaling data:
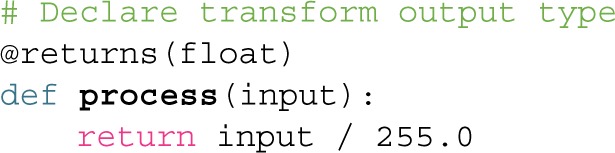
- NuGet: Bonsai modules are natively written in C# or other.NET languages. The NuGet package manager has emerged as the defacto standard for the sharing of code between.NET developers. Bonsai includes a full NuGet client which manages local package versions, provides access to the curated feed of standard Bonsai packages, and allows for the quick sharing of modules between Bonsai users through either NuGet or other remote and local package sources. Tutorials and examples on how to create new Bonsai modules are included in the Wiki.

## Results

### Architecture

Scientific data, like the world we live in, is inherently parallel. To monitor this complexity, modern experimenters are often forced to use multiple electronic instruments simultaneously, each with their own independent sampling rates. As data arrives at the acquisition computer, there are two main approaches to log and process these asynchronous data streams. The first approach is to use a polling strategy: a single sequential process in the computer runs a processing loop that goes through each device in sequence and gathers the available data. In this case, data from only one device is being collected and manipulated at any point in time. The second approach is to use an event-driven (reactive) architecture: processes are setup in parallel to collect data from all the devices simultaneously. Whenever new data is available, notifications are sent to the appropriate software routines that collect and process the data as soon as possible. When only a single processor is available, the difference between these two strategies is negligible: only one instruction at a time can be executed by the computer. However, with modern multi-processor cores and dedicated data transfer circuits, the performance difference between the two approaches will significantly influence the throughput of a data acquisition and processing system. Unfortunately, software tools to support and facilitate the “reactive” approach to data stream processing are only just now starting to be adopted and most software systems are still built from the sequential composition of simple program routines. Many of the assumptions of the sequential processing scenario do not scale to handle parallel execution, especially when shared memory and resources are involved.

In recent years, a number of advances in programming languages and software frameworks have tried to make it easier to create complex software applications by composition of asynchronous computing elements (Bainomugisha et al., 2013). Bonsai builds upon these new efforts and aims to extend these developments to the rapid-prototyping domain by introducing a visual programming language for composing and processing asynchronous data streams. Bonsai was developed on top of the Reactive Extensions for the.NET framework (Rx) (Microsoft Open Technologies, 2014). Rx provides many built-in operators that transparently deal with the concurrency challenges that inevitably surface when multiple data streams need to be processed and integrated together in a single program. It has become an increasingly popular framework to develop reactive interfaces for next generation mobile and desktop computing platforms, where it is used to handle the growing number of sensors and network communications required by business logic and consumer applications.

Bonsai (via Rx) represents asynchronous data streams using the notion of an observable sequence. An observable sequence represents a data stream where elements follow one after the other. An example would be a sequence of frames being captured by a camera, or a sequence of key presses logged by the keyboard. The name observable simply specifies that the way we access elements in the data stream is by listening to (i.e., observing) the data as it arrives, in contrast with the static database model, in which the desired data is enumerated.

**Figure 1:**
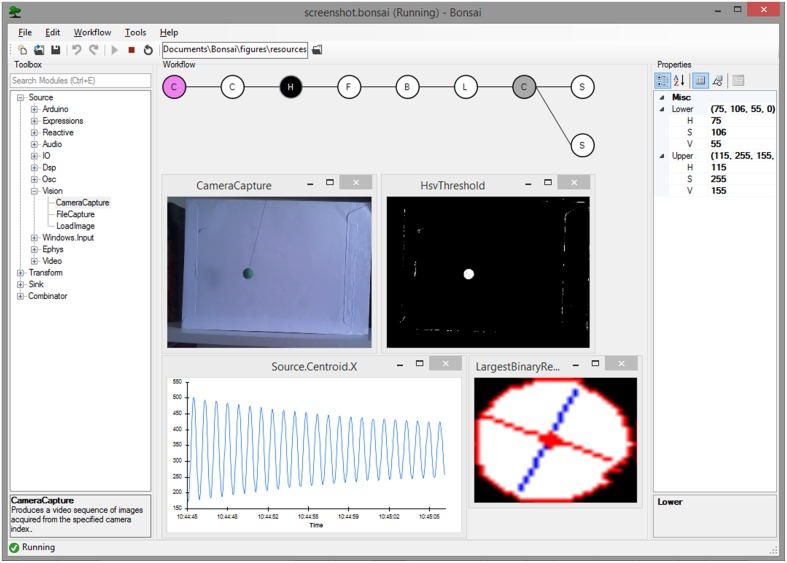
**Screenshot of the Bonsai user interface running a video processing pipeline**. An example dataflow for color segmentation and tracking of a moving pendulum is shown. Data sources are colored in violet; transform operators in white; sinks in dark gray. The currently selected node (Hsv Threshold) is colored in black and its configuration parameters are displayed in the properties panel on the right. Overlaid windows and graphs represent Bonsai data visualizers for the output of individual nodes.

**Figure 2:**
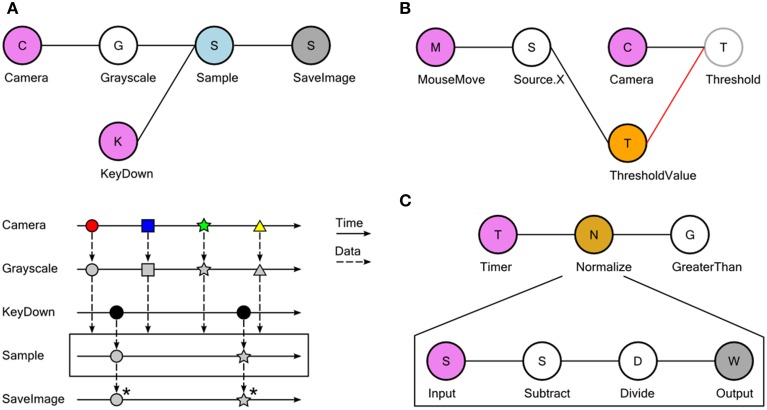
**Examples of dataflow processing pipelines using Bonsai**. **(A)** Taking grayscale snapshots from a camera whenever a key is pressed. Top: graphical representation of the Bonsai dataflow for camera and keyboard processing. Data sources are colored in violet; transform operators in white; combinators in light blue; sinks in dark gray. Bottom: marble diagram showing an example execution of the dataflow. Colored tokens represent frames arriving from the camera. Black circles represent key press events from the keyboard. Asterisks indicate saving of images to permanent storage. **(B)** Dynamic modulation of an image processing threshold using the mouse. The x-coordinate of mouse movements is used to directly set the externalized Threshold Value property (orange). The updated threshold value will be used to process any new incoming images. **(C)** Grouping a set of complex transformations into a single node. In the nested dataflow, the source represents incoming connections to the group and the sink represents the group output.

In Bonsai, observable sequences are created and manipulated graphically using a dataflow (Mosconi and Porta, 2000; Johnston et al., 2004) representation (Figures 1, 2A, Supplementary Video 1). Each node in the dataflow represents an observable sequence. Nodes can be classified as either observable sources of data or combinators (Table 1). Sources deliver access to raw data streams, such as images from a video camera or signal waveforms from a microphone or electrophysiology amplifier. Combinators represent any observable operator that handles one or more of these sequences. This category can be further specialized into transforms, sinks and other operator types depending on how they manipulate their inputs (Table 1). Transforms modify the incoming data elements of a single input sequence. An example would be taking a sequence of numbers and generating another sequence of numbers containing the original elements multiplied by two. Sinks, on the other hand, simply introduce processing side-effects without modifying the original sequence at all. One example would be printing each number in the sequence to a text file. The act of printing in itself changes nothing about the sequence, which continues to output every number, but the side-effect will generate some useful action. Combinators that change, filter or merge the flow of data streams are neither transforms nor sinks, and they are simply referred to by the more general term combinator. The *Sample* combinator illustrated in Figure 2A takes two data sequences and produces a new sequence where elements are sampled from the first sequence whenever the second sequence produces a new value. In this example, we use *Sample* to extract and save single images from a video stream whenever a key is pressed.

**Table 1:** **List of Bonsai node categories**.

**Color**	**Category**	**Description**
**BONSAI NODE CATEGORIES**		
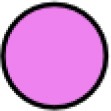	Source	**# Inputs: 0** Generates observable sequences of data
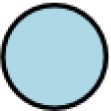	Combinator	**# Inputs: 0..N** Can change both the elements and the sequence; elements may be dropped, shifted, duplicated or merged with other sequences
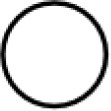	Transform	**# Inputs: 1** Transforms the elements in the sequence but does not change the sequence order or timing
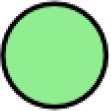	Condition	**# Inputs: 1** Does not change the elements in the sequence but can drop (filter out) elements from the sequence
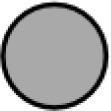	Sink	**# Inputs: 1** Does not change neither the elements nor the sequence, but can introduce side-effects
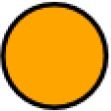	Property	**# Inputs: 1** Represents a property of another node. Writing to it at runtime will change the value of the property
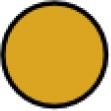	Nested	**# Inputs: 0..N** A nested combinator uses an internal (nested) dataflow to implement specific operations

*The color of each Bonsai node serves as a visual aid to identify their role in dataflow processing pipelines. Most of these categories are actually specializations of the very general combinator and are meant to visually depict their specific data processing semantics*.

A common requirement when designing and manipulating dataflows is the ability to visualize the state of the data at different stages of processing. We have therefore included a set of visualizers to assist debugging and inspection of data elements, including images and signal waveforms (Figure 1). These visualizers are automatically associated with the output data type of each node and can be launched at any time in parallel with the execution of the dataflow. Furthermore, it is often desirable to be able to manipulate processing parameters online for calibration purposes. Each node has a set of properties which parameterize the operation of that particular source or combinator (Figure 1). This allows, for example, changing the cutoff frequency of a signal processing filter, or setting the name of the output file in the case of data recording sinks. We have also included the possibility of externalizing node properties into the dataflow (Figure 2B). Externalizing a property means extracting one of the parameters into its own node in the dataflow, making it possible to connect the output of another node to the exposed property. This allows for the dynamic control of node parameters.

Finally, we have built into Bonsai the ability to group nodes hierarchically. In its simplest form, this feature can be used to encapsulate a set of operations into a single node which can be reused elsewhere (Figure 2C). This is similar to defining a function in a programming language and is one of the ways to create new reactive operators in Bonsai. Any named externalized properties placed inside an encapsulated dataflow will also show up as properties of the group node itself. This allows for the parameterization of nested dataflows and increases their reuse possibilities. In addition, encapsulated dataflows are used to specify more complicated, yet powerful, operators such as iteration constructs that allow for the compact description of complex data processing scenarios that can be cumbersome to specify in pure dataflow visual languages (Mosconi and Porta, 2000) (see below).

Bonsai was designed to be a modular framework, which means it is possible to extend its functionality by installing additional packages containing sources and combinators developed for specific purposes. New packages can be written using C# or any of the.NET programming languages. Python scripts [via IronPython (IronPython Community, 2014)] can be embedded in the dataflow as transforms and sinks, allowing for rapid integration of custom code. All functionality included in Bonsai was designed using these modular principles, and we hope to encourage other researchers to contribute their own packages and thereby extend the framework to other application domains. At present, the available packages include computer vision and signal processing modules based on the OpenCV library (Itseez, 2014). Drivers for several cameras and interfaces to other imaging and signal acquisition hardware were integrated as Bonsai sources and sinks, including support for Arduino microcontrollers (Banzi et al., 2014), serial port devices and basic networking using the OSC protocol (Wright et al., 2003). Given the specific applications in the domain of neuroscience, we also integrated a number of neuroscience technology packages. The Ephys package, for example, builds on the Open Ephys initiative for the sharing of electrophysiology acquisition hardware (Voigts et al., 2013) by providing support for the Rhythm open-source USB/FPGA interface (Intan Technologies, US). Therefore, the next generation tools for electrophysiology can already be used inside Bonsai, the acquired physiology data implicitly integrated with other available data streams and thus easily assembled into a powerful and flexible experimental neuroscience platform.

### Advanced operators

**Figure 3:**
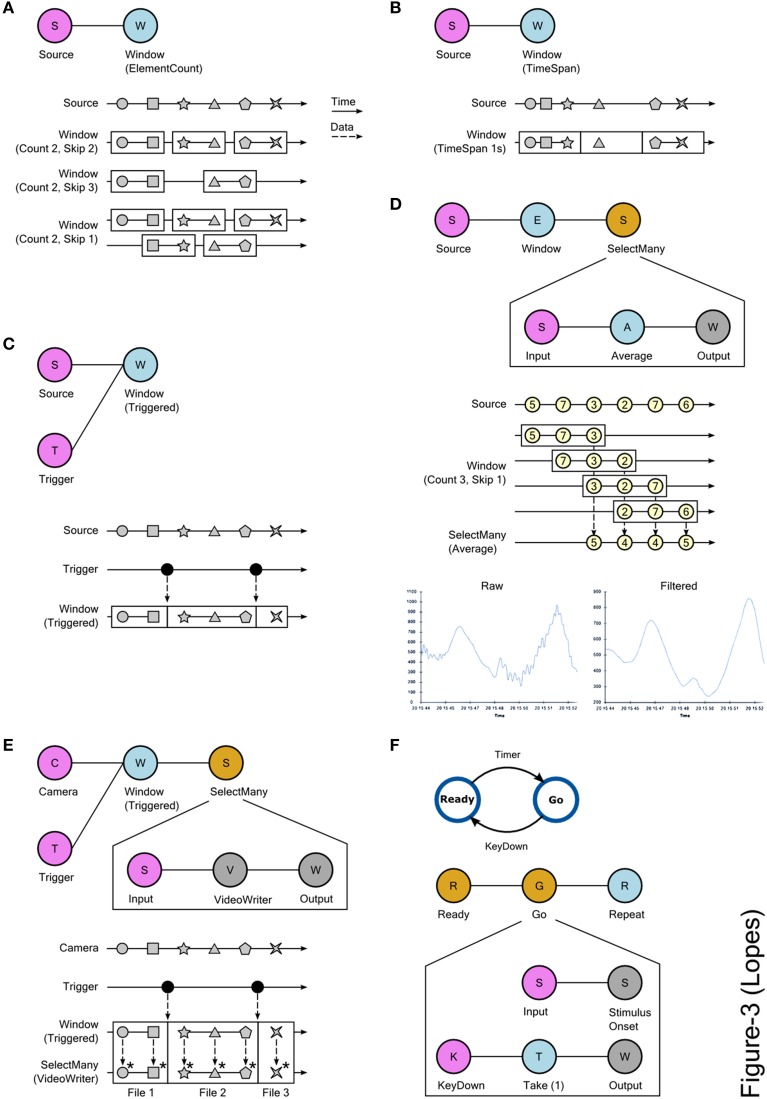
**Using slicing and window processing combinators in Bonsai**. **(A)** Creating data windows using element count information. The Count and Skip parameters specify the size of each window and the number of elements to skip before creating a new window, respectively. Top: graphical representation of the Bonsai dataflow used for slicing. Bottom: marble diagram showing the behavior of the operator for different values of the parameters. The boundaries of each window are indicated by the enclosing rectangles. **(B)** Creating data windows using timing information. Time is split into intervals of equal fixed duration. Each interval defines a window and data elements are assigned to each window based on the interval that is active at the time of their arrival. Top: Bonsai dataflow. Bottom: marble diagram. **(C)** Creating data windows using an external trigger. The boundaries of the created windows are defined by the timing of events produced by the trigger source. Top: Bonsai dataflow. Bottom: marble diagram. **(D)** Moving average of a signal source using windows. Sliding windows of the data are created based on element count information. Top: Bonsai dataflow. The dataflow encapsulated in *SelectMany* specifies the processing done on each window. In this case, the average value of each window sequence is computed. Middle: marble diagram. As soon as each window is completed, its average value is merged into the result sequence. Bottom: example signal trace before and after the filtering. **(E)** Online splitting of video recordings into different files based on an external trigger. Top: Bonsai dataflow. Notice that the *VideoWriter* sink is included inside the *SelectMany* combinator. Bottom: marble diagram. At the start of each window, a new movie file is created. Asterisks indicate the encoding of individual frames in each window to the corresponding file. **(F)** Implementing state-machines using window operators. Top: state-machine schematic of a task designed to measure response times. In the Ready state, the stimulus is off. When entering the Go state, the stimulus is turned on. At the end of each trial, the system goes back to the initial state. Bottom: graphical representation of the equivalent Bonsai dataflow. The *SelectMany* combinator is used to specify the behavior and transitions of each state. The *Take* combinator truncates a sequence to include only a specified number of initial elements. In this case, only the first element is included. The *Repeat* combinator restarts a sequence when no more elements are produced (see text).

The most common application of Bonsai is the acquisition and processing of simple, independent data streams. However, for many modern experiments, basic acquisition and storage of data is often not sufficient. For example, it can be convenient to only record the data aligned on events of interest, such as the onset of specific stimuli. Furthermore, neuroscience experiments often progress through several stages, especially for behavioral assays, where controlled conditions vary systematically across different sessions or trials. In order to enforce these conditions, experiments need to keep track of which stage is active and use that information to update the state of control variables and sensory processing. These requirements often cannot be described by a simple linear pipeline of data, and require custom code to handle the complicated logic and bookkeeping of experimental states. Below we describe a set of advanced Bonsai operators that can be used to flexibly reconfigure data processing logic to cover a larger number of scenarios. These operators and their applications are all built on the single idea of slicing a data stream into sub-sequences, called windows, which are then processed independently and, potentially, in parallel (Figure 3).

Bonsai provides different combinators that allow the creation of these sub-sequences from any observable data stream, using element count information, timing, or external triggers (Figures 3A–C). The specific set of operations to apply on each window is described by encapsulating a dataflow inside a *SelectMany* group, as detailed in the signal processing example of Figure 3D. The input source in this group represents each of the window sub-sequences, i.e., it is as if each of the windows is a new data source, containing only the elements that are a part of that window. These elements will be processed as soon as they are available by the encapsulated dataflow. Windows can have overlapping common elements, in which case their processing will happen concurrently. The processing outputs from each window are merged together to produce the final result. In the case of Figure 3D, past and future samples are grouped in windows to compute a running average of the signal through time, necessarily time-shifted by the number of future samples that are considered in the average.

The processing of the elements of each window happens independently, as if there was a new isolated dataflow running for each of the sequences. We can exploit this independence in order to dynamically turn dataflows on and off during an experiment. In the video splitting example of Figure 3E, we use an external trigger source to chop a continuous video stream into many small video sequences, aligned when the trigger fired. We then nest a *VideoWriter* sink into the *SelectMany* group. The *VideoWriter* sink is used to encode video frames into a continuous movie file. It starts by creating the video file upon arrival of the first frame, and then encoding every frame in the sequence as they arrive. When the data stream is completed, the file is closed. By nesting the *VideoWriter* inside the *SelectMany* group, what we have effectively done is to create a new video file *for each* of the created windows. Whenever a new trigger arrives, a new clip is created and saving proceeds, implicitly parallelized, for that video file.

More generally, we can use this idea to implement discrete transitions between different processing modes, and chain these states together to design complex control structures such as finite state machines (FSMs). FSMs are widely used to model environments and behavioral assays in systems and cognitive neuroscience. One example is illustrated in Figure 3F, where we depict the control scheme of a stimulus-response apparatus for a simple reaction time task. In this task, there are only two states: Ready and Go. In the Ready state, no stimulus is presented and a timer is armed. Whenever the timer fires, the task transitions into the Go state, and a stimulus is presented. The subject is instructed to press a key as fast as possible upon presentation of the stimulus. As soon as the key is pressed, the system goes back to the Ready state to start another trial. In a FSM, nodes represent states, e.g., stimulus availability or reward delivery, and edges represent transitions between states that are caused by events in the assay, e.g., a key press. In each state, a number of output variables and control parameters are set (e.g., turning on a light) which represent the behavior of the machine in that state.

In the Bonsai dataflow model, dataflows encapsulated in a *SelectMany* group can be used to represent states in a FSM (Figure 3F, bottom). Specifically, a state is activated whenever it receives an input event, i.e., the dataflow nested inside the state will be turned on. The dynamics of the nested dataflow determine the dynamics of the state. In the Go state presented in Figure 3F, the activation event is used to trigger stimulus onset. In parallel, we start listening for the key press which will terminate the state. Conversely, for the Ready state we would trigger stimulus offset and arm the timer for presenting the next stimulus. An important difference between Bonsai dataflows and pure state machine models is that a dataflow is specified as a directed acyclic graph, i.e., the data stream cannot loop back on itself. However, by taking advantage of the *Repeat* combinator, we can restart a dataflow once it is completed, allowing us to reset the state machine for the next trial.

Many of the control tasks in experiments have this sequential trial-based structure, which has allowed us to rapidly prototype complex behavior assays, such as closed-loop rodent decision making tasks, simply by leveraging the flexibility of the data stream slicing operators.

### Alternatives to bonsai

Although graphical user interfaces have played a crucial role in the widespread proliferation of computing technology throughout various scientific fields, the majority of these interfaces tend to be applied to relatively narrow domains, such as the operation of a specific instrument. Their goal is often to provide access to all the various configuration parameters of the hardware and to provide basic data acquisition functionality. There is often no opportunity to parameterize or condition the behavior of the instrument beyond the possibilities presented by the interface, and interconnections with other devices are often limited to simple hardware triggers. The alternative, when available, is to access low-level application programming interfaces (APIs), and program the desired behavior from scratch.

In the more flexible domains of data analysis, behavior control and software simulations, the use of more versatile graphical interfaces has become increasingly prevalent. In these scenarios, it is not uncommon to encounter the development of domain-specific languages (DSLs), where graphical building blocks related to the domain of application can be combined together by the user to generate new behaviors, such as the sequence of steps in a psychophysics experiment or a state-machine diagram used to control stimuli and rewards in operant conditioning. While providing more flexibility to the end user, such DSLs are usually not conceived, at their core, to be applied to wildly different domains (e.g., an operant conditioning state machine is not expected to be able to filter continuous electrophysiology signals). In fact, most DSLs will not even allow the user to extend the set of built-in operations. In those that do, the developer may find a customization pit (Cook et al., 2007), where concepts and operations that are within the range of what the DSL can express are easy to develop, whereas tasks that are a little bit outside of the boundaries of the language quickly become impossible or too cumbersome to implement.

As the level of flexibility of a graphical user interface increases, we start to approach the space occupied by general purpose visual programming languages (GPVPL). These are languages that are designed from the outset to be capable of solving problems across a wide variety of domains using a general set of operations. Ideally, the core building blocks of the language will themselves be domain-independent, so that the user can easily apply the same set of operations to the widest possible class of inputs. In order to better illustrate the feel and expressive power of GPVPLs, and to clarify where Bonsai itself is positioned, we will give two examples of popular languages that have succeeded in this niche: LabVIEW (National Instruments, 2014) and Simulink (MathWorks, 2014).

LabVIEW is one of the best examples of a GPVPL applied to the design and control of experiments (Elliott et al., 2007). In LabVIEW, users create virtual instruments (VIs) which are composed of a graphical front-panel containing an assortment of buttons, dials, charts and other objects; as well as a back-panel where a flowchart-like block diagram can be used to specify the behavior of the VI. In this back-panel, nodes and terminal elements can represent hardware components, numerical operations or front-panel objects, which are connected together using virtual wires that specify the flow of data between them. The popularity of LabVIEW grew initially from its support for state-of-the-art data acquisition cards and hardware as well as its data visualization capabilities. The modularity of its architecture also allowed users to quickly develop and implement new nodes within the language itself by using VIs themselves as nodes.

Although the LabVIEW back-panel is a dataflow visual programming language, its execution model tends to follow a polling, rather than event-driven, strategy for dealing with multiple data streams. In order to properly scale this model to the increasing number of available processor cores, LabVIEW has implemented sophisticated code analysis tools that attempt to identify parallelizable portions of block diagrams automatically (Elliott et al., 2007). Once these sections are identified, LabVIEW will automatically generate parallel processes depending on the number of available cores and will manage the bottlenecks in the code accordingly. Although this mitigates the limitations of the sequential polling programming model, it is important to realize that the goal of such automatic parallelization is still to provide the user with a logically synchronized programming model.

Simulink is a popular dataflow visual programming language for modeling, simulating and analyzing multi-domain dynamic systems. It has become extremely popular for modeling response characteristics of control systems, allowing not only for the rapid prototyping of algorithms, but also the automatic generation of microcontroller code for embedded systems. Again, the success of the language stemmed primarily from the flexibility and ease of use of the block diagrams, as well as the number of prebuilt operations and data visualization tools which quickly took care of many crucial but tedious aspects of control systems modeling.

Like LabVIEW, the execution model for Simulink generated code is still based on polling strategies, where ready to execute dataflow nodes are updated in turn as inputs become available. Again, strategies to scale the output of Simulink to multiple cores have been proposed based on analyzing and segmenting the model into parallelizable sections which can be converted into equivalent parallel execution code for microcontrollers (Kumura et al., 2012).

Similar to LabVIEW and Simulink, Bonsai was designed as a general purpose modular language. The core architecture of Bonsai is domain-independent and provides a general framework to compose asynchronous data streams. A general set of composition operators, or combinators, provides support for iteration, segmentation and merging of parallel data streams, as well as other common manipulations on observable sequences. Both the sources of data and available processing operations can be extended within the language itself using nesting of dataflows. Data visualizers and a growing library of data stream acquisition, processing and logging modules are provided to allow rapid prototyping of a large number of different applications.

However, in contrast to LabVIEW or Simulink, Bonsai adopts a very different strategy to implement dataflow execution. Rather than trying to derive a global sequential execution order of dataflow nodes based on the number of active inputs, Bonsai nodes simply react to incoming inputs immediately, without the need to wait for all of them to be active. When multiple observable sequences are present, this allows for a choice of different concurrency composition strategies. Nevertheless, as the result of the composition is an observable sequence itself, such concurrency management can remain functionally isolated from the combinator that is handling the composition. From the point of view of downstream operators, they are simply receiving an observable sequence. There is a tradeoff, of course, that more responsibility for managing the flow of data is passed to the end user, but it also allows for a finer grained control of concurrency that is critical to the specification of parallel applications.

One important caveat of developing asynchronous systems is that debugging can be more difficult in situations where the precise timing and ordering of events is required to reproduce an offending behavior. In synchronized and sequential execution environments, one can easily go step by step through the precise cascade of transformations that resulted in a problem. In contrast, when multiple processes are executing concurrently, it can be harder to analyze the program flow in a similarly reproducible, deterministic manner. However, it should be noted that this issue is not unique to reactive environments with real asynchronous devices. A sequential polling strategy will be equally deficient in reproducing a particular execution sequence when data from parallel input devices is being accessed.

Another important caveat is that Bonsai currently runs exclusively in Windows operating systems. However, Microsoft has recently open-sourced the execution engine of the.NET framework and will pursue implementations for all the major operating systems (Linux/Mac). This raises the interesting possibility of eventually extending the Bonsai user base into these important platforms.

### Applications

**Figure 4:**
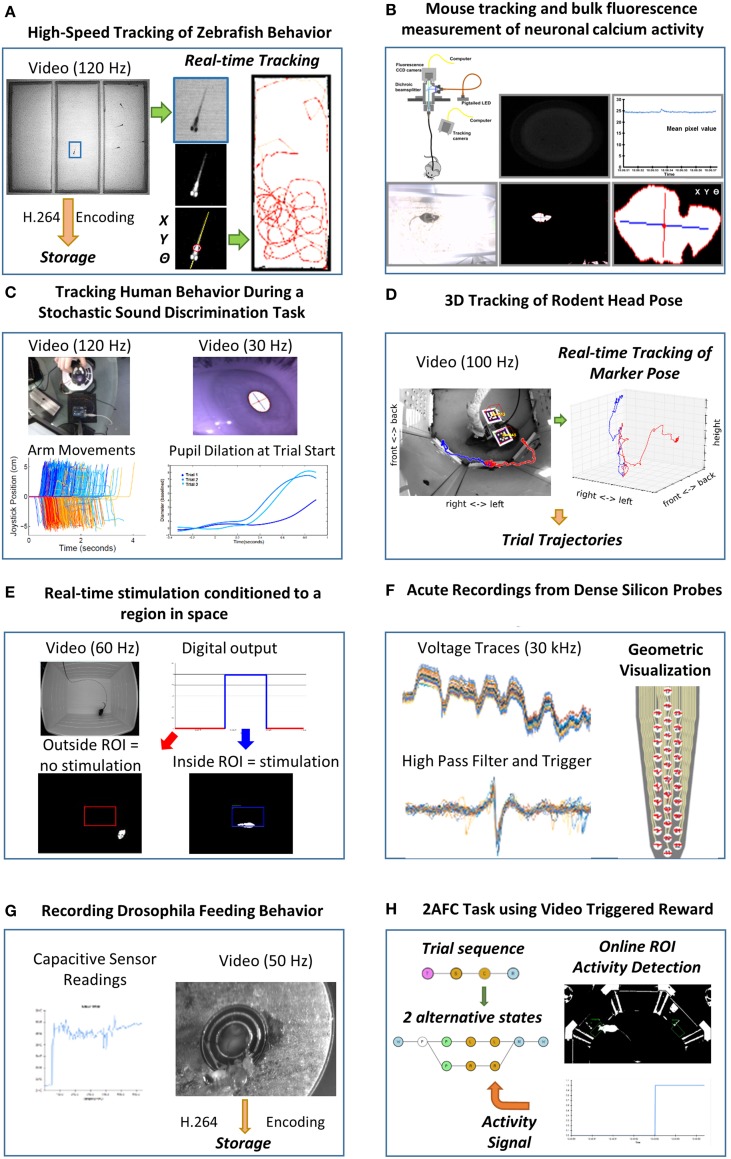
**Example use cases of neuroscience experimental setups using Bonsai**. **(A)** High-speed tracking of zebrafish behavior. Insets depict the image processing steps for segmenting the shape of a fish from the background and extracting its spatial location and orientation. Right: example trajectories extracted from an individual fish. **(B)** Mouse tracking and bulk fluorescence measurement of neuronal calcium activity. Top insets: schematic of the fiber optic imaging setup for freely moving rodents with example fluorescence data frame and extracted fluorescence signal traces. Bottom insets: image processing steps for behavior tracking of a mouse as the largest dark object in the video. **(C)** Tracking human behavior during a stochastic sound discrimination task. Left insets: arm movements on the joystick on each trial tracked by brightness segmentation of a bright LED. Right insets: extraction of pupil dilation by computing the length of the major axis of the largest dark object. **(D)** 3D tracking of rodent head pose. Left inset: example video frame of a mouse carrying fiducial markers. A cube was rendered and superimposed on the image to demonstrate correct registration. Colored traces show representative single trial trajectories of an individual marker, aligned on center poke onset. Red and blue refer to left and right choice trials, respectively. Right inset: Three-dimensional plot of the same trajectories using isometric projection. **(E)** Real-time stimulation conditioned to a region in space. Top insets: example raw movie frame and stimulation state. Red and blue indicate no stimulation and stimulation regimes, respectively. Bottom insets: example video frames where the mouse is either outside or inside the region of interest. **(F)** Acute recordings from dense silicon probes. Left insets: example traces from raw amplified voltage signals and high-pass filtered spike triggered waveforms. Right inset: visualization of spike waveforms triggered on a single channel superimposed on the actual probe geometry. **(G)** Recording *Drosophila* feeding behavior. Left inset: example trace of a single-channel capacitive signal from the flyPAD. Right inset: simultaneously recorded video of the fly feeding behavior. **(H)** 2AFC task using video triggered reward. Left inset: schematic of the reactive state machine used for controlling the task. Each state is represented by a nested dataflow. Branches represent possible transitions. Right inset: example thresholded activity from a single region of interest activated by the mouse.

The validation of Bonsai was performed by using the framework to implement a number of applications in the domain of neuroscience (Figure 4). The breadth of technologies at use in this field demands that modern experiments be able to handle many heterogeneous sources of data. Experimenters need to routinely record video and sensor data monitoring the behavior of an animal simultaneously with electrophysiology, optical reporters of neural activity or other physiological measures. Online manipulation and visualization of data is a fundamental part of the experiment protocol for many of the reported techniques. In the following, we highlight some of these practical applications of Bonsai in more detail in order to illustrate both “best practices” and implementation strategies.

One of the first use cases driving the development of Bonsai was the automated online tracking of animal behavior using video. The most common tracking application involves chaining together operators for image segmentation and binary region analysis to allow the extraction of the spatial location of an animal over time (Figures 4A,B). The same technique can easily be extended to track different kinds of objects, such as eyes or experimental manipulanda in human psychophysics experiments (Figure 4C), provided adequate illumination contrast and the appropriate choice of a method for segmentation. These image processing tools can also be used to acquire and process physiological data in neural imaging setups, where it is now possible to record bioluminescent or fluorescent reporters of neural activity during behavior. For example, Figure 4B demonstrates simultaneous measurement of animal behavior and neural activity using bulk fluorescence calcium imaging in the mouse brain recorded with a CCD sensor and a fiberoptic system (Tecuapetla et al., 2014).

Raw video data from modern high-resolution, high-speed cameras can be expensive and cumbersome to store. Online video compression and storage sinks were implemented taking advantage of parallelism to avoid frame loss. Video compression is processing intensive and can compromise data acquisition if reading the next frame has to wait for the previous frame to be fully encoded. One solution is to buffer incoming frames and compress them in parallel with the rest of the processing stream. By encapsulating this behavior into a Bonsai sink, it became easy to incorporate video recording and compression functionality into any image processing pipeline (Figures 4A–E,G,H).

While simple image processing techniques can easily extract continuous two-dimensional measures of animal location over time, it often becomes the case that the experimenter is concerned with tracking the detailed behavior of specific features in the animal's body, such as head pose. This is an essential component in neurophysiology or stimulation experiments in freely moving animals, where ongoing behavior is the central constraint in interpreting neural responses and manipulations. However, identifying such features and reconstructing their position and orientation in 3D space is a challenging computer vision problem. A common solution is to use planar fiducial markers of known geometry (Kato and Billinghurst, 1999; Garrido-Jurado et al., 2014) (Figure 4D). The computer vision research community has developed some open-source software solutions to this problem (Garrido-Jurado et al., 2014), which have been integrated into Bonsai to allow the possibility of easily and flexibly incorporating online 3D fiducial tracking in video streams. This approach has been used successfully to record 3D head movements of a mouse under optogenetic stimulation in a decision-making task (Figure 4D).

One final, but important application of video stream processing is in the development of closed-loop interfaces, where the actions of an animal directly modulate manipulations under the experimenter's control. This kind of experiment requires fast online analysis of behavior and physiological variables of interest that are subsequently coupled to hardware control interfaces. In Figure 4E, real-time stimulation conditioned to a region in space was implemented by analyzing the position of an animal in a square arena. Whenever the animal found itself inside a specified region of interest, a signal was sent to an Arduino controller which was then used to drive optogenetic stimulation of specific neural circuits.

Another key data type that is commonly processed by Bonsai dataflows is buffered time-series data. This type of data usually arises from audio, electrophysiology or other digital acquisition systems where multiple data samples, from one or more channels, are synchronously acquired, buffered and streamed to the computer. These buffers are often represented as data matrices, where rows are channels and columns represent individual data samples through time, or vice-versa. Support for simple band-pass filters, thresholding and triggering allowed us to build flexible spike detection and waveform extraction systems (Figure 4F). Using Intan's Rhythm API, we integrated into Bonsai support for a variety of next-generation electrophysiology devices using Intan's digital amplifier technology, such as the Open Ephys acquisition system (Voigts et al., 2013) or Intan's evaluation board (RHD2000, Intan Technologies, US). This system was successfully used to acquire and visualize simultaneous recordings from dense silicon probes where spikes from a loose-patch juxtacellular pipette were used as triggers to align and extract waveform data appearing on the multi-channel extracellular probe. Responses from every silicon probe site could then be superimposed on an accurate rendition of the probe geometry, in real-time.

The ability to rapidly integrate new modules allowed us to support the development and cross-validation of new tools for behavioral neuroscience. A paradigmatic example was the flyPAD, a new method for quantifying feeding behavior in *Drosophila* melanogaster by measuring changes in electrode capacitance induced by the proboscis extension of a fly (Itskov et al., 2014). The integration of the flyPAD in Bonsai allowed researchers to quickly get started using this approach to design new experiments. Furthermore, it also allowed the validation of the tool by enabling simultaneous acquisition of high-speed video recordings of fly behavior which were later used for annotation and classification of the sensor feeding traces (Figure 4G).

In a different set of experiments, Bonsai was used to implement a variation on a popular two-alternative forced choice (2AFC) decision-making task for rodents (Figure 4H). In this type of task, animals are placed in an environment with three “ports.” They are presented with a stimulus in the center port and afterwards report their perception of the stimulus by going either to the left or right choice ports. In the variation we present in this work, the two choice ports were replaced by regions of interest where the activity of the animal is analyzed using computer vision. This example offered unique challenges as it combined sophisticated sequential control of a task environment with continuous data stream processing of video and sensor data.

The integration of all these diverse components for data acquisition and experiment control does not only allow for the rapid deployment of established protocols. In fact, the modular nature of their integration (i.e., how they can be combined together) opens up new avenues for research, by allowing a rich, rapid exploration of novel methodologies. To demonstrate this, we created a dynamic virtual environment for freely moving rodents where the visual presentation of a stimulus is tightly controlled in closed-loop to the actions of the animal. We used a projection setup similar to the low-cost multi-touch sensing table proposed by Han (2005), where a visible light rear-projection system is coupled with infrared illumination and an infrared imaging sensor to detect in real-time where the animal is located with respect to the visual display surface (Supplementary Video 2).

## Discussion

After about a year of using Bonsai in an active neuroscience research institute, dozens of different experimental protocols and data analysis pipelines have been successfully implemented using the provided building blocks (Gouvêa et al., 2014; Itskov et al., 2014; Tecuapetla et al., 2014). We were surprised by the diversity of applications and by the pace at which new modules and devices were developed and integrated.

The performance achieved by Bonsai dataflow processing was an important consideration throughout (Box 2). Video processing can be particularly challenging to handle given the bandwidth required to quickly acquire and process large data matrices. In order to correlate continuous measures of behavior with neural activity, it is useful for those measurements to have both high spatial and high temporal resolution. Using Bonsai, we were able to simultaneously process and compress grayscale image sequences from high resolution (1280 × 960) and high frame rate (120 Hz) cameras using standard off-the-shelf desktop computers (Intel Core i7, 8 GB RAM). In fact, many of the reported assays use multiple (>2) such video streams with success and actually process the behavior video online either to control states of the behavior protocol or to pre-process video data for offline analysis.

Box 2 **Under the Hood**

**Computational Overhead**

Bonsai takes full advantage of the flexibility of C# and its Just-In-Time (JIT) compiler to bring the computational overhead of running the framework to zero. This is possible due to the fact that the graphical dataflows in Bonsai are actually specifying syntactically correct C# code by means of an expression tree. When the dataflow is executed, C# code is generated for assembling and running the pipeline. This code is ultimately compiled into native machine language before execution, which has the consequence that running a Bonsai dataflow is as fast as if one wrote the equivalent Rx code manually. In fact, this also means every Bonsai module is just a standard C# class exposing methods working on Rx's observable interface, which makes it possible to reference every single Bonsai package from a standard.NET application and just use the module functionality directly.

**Concurrency**

The level of concurrency and parallelism in Bonsai entirely depends on the structure of each individual dataflow and the specific computer hardware involved. Typically, each hardware device source (e.g., a camera) runs independently in its own logical thread. Some sources can occasionally share threads when the underlying device architecture allows for it. For example microcontroller sources coming from the same USB port effectively require sharing a single communications channel, but this is logically abstracted from the developer so there is no need to worry about handling multiplexed messages.

The specialized handling of concurrency introduced by merging different processing streams is done using dedicated Rx concurrency operators that are exposed graphically through the language. Operators located downstream from the merge point can treat the merged sequence as if it was a single sequential data source. This means most Bonsai operators are actually concurrency-agnostic, meaning they don't have to worry about concurrency at all: they simply assume their inputs are processed sequentially. This functional approach allows Bonsai operators to be simple to program, reliable and extremely performant.

Finally, some Bonsai operators introduce local concurrency implicitly to maximize performance. For example, many of the data logging sinks actually write to disk in parallel with the arrival of data. This prevents processor-heavy routines, such as video compression, to stall the pipeline and allow for online execution to proceed as fast as possible. From the point of view of the developer, however, such optimizations happen transparently.

**Time**

Being a fully asynchronous framework, Bonsai has to deal with code executing logically in many different processors. There is no particular assumption about time in the framework other than the sequential flow of data through the pipeline, but facilities are in place to help the synchronization and timing of data. For example, the *Timestamp* operator provides access to hardware performance timers included in modern processors to timestamp event notifications, across the pipeline, using a shared high resolution clock. However, it should be noted that this only applies to processes occurring centrally: for precise sub-millisecond synchronization of physical events happening outside the computer (e.g., stimulation pulse train and electrophysiology data) we still recommend the classical sharing of voltage or optical sync pulses logged simultaneously in each device.

**Figure 5:**
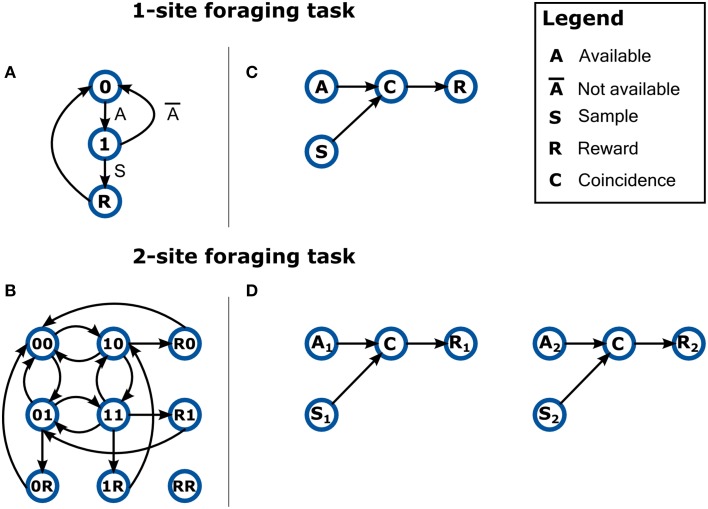
**Describing the behavior of dynamic environments using either state-machines or dataflows**. **(A)** A state-machine model of the 1-site foraging task. Zero indicates non-availability of reward at the site. One indicates reward is now available at the site. Labels on edges indicate event transitions. **(B)** A non-exhaustive state-machine model for a foraging task with two sites. The active state is now a combination of the state of the two sites (indicated by a two character label) and all possible state combinations are tiled across the model. Event labels are omitted for clarity. Notation is otherwise kept. **(C)** A dataflow model of the 1-site foraging task. Events in the state-machine model are now modeled as data sources. The coincidence detector node propagates a signal only when the sample event closely follows reward availability. **(D)** A dataflow model for a foraging task with two sites. The number subscripts denote foraging site index.

One of the areas where we see the application of Bonsai becoming most significant is in the development of dynamic behavior assays (environments) using reactive control strategies. Brains evolved to generate and control behaviors that can deal with the complexity of the natural world. However, when neuroscientists try to investigate these behaviors in the lab, it is often difficult to design equivalent environmental complexity in a controlled manner. As an example, consider a simple foraging scenario in which a land animal must collect, in a timely manner, food items that become available at random intervals in many sites. If the item is not collected in time, it rots or gets eaten by competitors. In the case of a single foraging site, a FSM description intuitively represents the workings of the environment (Figure 5A). However, let us now consider a situation where the environment has two of these food sites operating independently, thus introducing the possibility of different events occurring simultaneously at each of the sites. If our environment is modeled as a finite-state machine, then we must represent every possible combination of states and transitions, as in Figure 5B. In the classical state machine formalism the machine can only be in one state at a time, which means we now need to model each state as the combination of the individual independent states at each reward location. Furthermore, because transitions between these states are asynchronous and independent, we thus have edges between nearly every pair of nodes, as each reward site can change its state at any point in time relative to the other.

How would designing such a scenario feel like in a reactive programming language? Figure 5C shows a possible specification of the 1-site foraging task in reactive terms. In this case, we have two sources of events from the environment: one timer signaling the availability of reward (A); and a sampling event (S) which is triggered every time the animal checks the location for food. Both of these events can occur independently of each other, but when a sampling event coincides with reward availability (C), then reward (R) is delivered. Because this description is intrinsically asynchronous and parallel, it makes it extremely easy to scale the task to a larger set of locations: just replicate the dataflow for each of the other locations (Figure 5D). In this example, the design space was made more intuitive by introducing the parallel and asynchronous nature of a real-world situation into our modeling formalism.

Another difficulty of the classical state machine formalism is dealing with continuous variables. The natural environment provides constant real-time feedback that tightly correlates with the actions of an animal. Reproducing such closed-loop interaction and manipulating its dynamics is a necessary tool for fully investigating brain function. Such models are virtually impossible to represent in a machine of finite states, given the potential infinitude of feedback responses. However, the dataflow formalism of asynchronous event sources can easily accommodate such models. In fact, this is their natural battleground; nodes represent reactive operators that promptly respond to input values broadcasted by event sources. These models of asynchronous computation are thus ideal for recreating the complex discrete and continuous aspects of natural environments that brains evolved to master. We thus propose Bonsai as a new tool for neuroscientists trying to understand how the brain deals with real world complexity.

## Materials and methods

All experiments were approved by the Champalimaud Foundation Bioethics Committee and the Portuguese National Authority for Animal Health, Direcção-Geral de Alimentação e Veterinária (DGAV).

### High-speed tracking of zebrafish behavior

Larval zebrafish (6 dpf) were filmed with a high-speed monochrome video camera (Flea3, Point Gray, CA) under IR illumination. Fish swam freely in a custom-built arena that was laser cut from transparent acrylic that consisted of three separate chambers, each 40 × 100 mm. The position and orientation of the zebrafish in the central chamber was continuously tracked in real-time, while the video of the entire arena (1.3 Megapixel) was compressed to a high-quality H.264 encoded file that was used for subsequent offline analysis of the interaction between individuals and groups of zebrafish placed in either of the side chambers.

### Mouse tracking and bulk fluorescence measurement of neuronal calcium activity

Freely behaving mice were filmed with a video camera (PlayStation Eye, Sony, JP) under white light illumination in their own homecages. A fiberoptic setup was developed to monitor bulk fluorescence changes in specific neuron populations using genetically encoded calcium indicators. Changes in fluorescence caused by neuronal activity were transmitted by an optical fiber and recorded with a CCD camera (Pike, Allied Vision Technologies, DE). The position and orientation of the mice was continuously tracked in real-time, while the mean pixel value of the area of the camera facing the fiber optic was continuously calculated. Both videos were compressed to high-quality H.264 encoded files to be used in subsequent offline analysis.

### Tracking human behavior during a stochastic sound discrimination task

Bonsai was used to acquire timestamped images from three cameras (eye, person's view, and arm) simultaneously. The videos are synced with presented sound stimulus by using the arm camera to also capture two IR LEDs which are turned on at sound on-set and off-set, respectively. The arm is tracked by using an IR LED mounted on a joystick and processing the video online at 120 Hz to minimize noise from compression. All the videos are compressed to a MP4 encoded file for offline analysis of the eye movements, pupil dilation, and syncing of all events with the sounds. The eye videos are captured at 30 Hz using the IR illuminated pupil headset (https://code.google.com/p/pupil/).

### 3D tracking of rodent head position

Adult mice performing a two alternative forced choice task were filmed with a high-speed monochrome video camera (Flea3, Point Gray, CA). A fiber optic cable was attached to the mouse's head. The 3D position and orientation of the head was tracked in real-time using square fiducial markers from the ArUco tracking library (Garrido-Jurado et al., 2014). The video (0.24 Megapixel) was simultaneously compressed to a high-quality H.264 encoded file that was used for subsequent offline analysis of the behavior.

### Real-time stimulation conditioned to a region in space

Black mice were recorded with a high speed video camera (Flea3, Point Gray, CA), while exploring an open field arena (50 × 40 cm, L × W), under white illumination (~250 lux). The x and y position, body centroid and orientation of the animal in the arena was continuously tracked in real-time. Mice were implanted with an optical fiber connected to a laser, in order to receive photostimulation with blue light. A region of interest (ROI, 13 × 10.5 cm, L × W) was defined and a python script was written to outline the conditioning protocol. A digital output signal was sent to a microcontroller board (Uno, Arduino, IT), each time the body centroid of the animal entered in the ROI, producing photostimulation. All data for the animal tracking and digital output was saved in a.csv file, as well as the video, for subsequent offline analysis of the behavior.

### Acute recordings from dense silicon probes

Recordings of spontaneous neural activity in motor cortex were performed in anesthetized rodents by means of silicon probes comprising a dense electrode array (A1x32-Poly3-5 mm-25s-177-CM32, Neuronexus, US). An open-source electrophysiology acquisition board (Open Ephys) was used along with a RHD2000 series digital electrophysiology interface chip that filters, amplifies, and digitally multiplexes 32 electrode channels (Intan Technologies, US). Extracellular signals sampled at 30 kHz with 16-bit resolution in a frequency band from 0.1 to 7500 Hz were saved into a raw binary format for subsequent offline analysis. Online analysis of neural spike waveforms across all probe channels was performed by aligning the multi-channel raw data on spike events from a selected channel of interest. A custom Bonsai visualizer was written using OpenGL to display all channel traces superimposed on the geometric arrangement of probe sites. It was possible to examine the details of extracellular activity in the spatial distribution.

### Recording *Drosophila* feeding behavior

Individual *Drosophila* melanogaster flies were allowed to freely feed on the flyPAD (Itskov et al., 2014) while their feeding behavior was monitored at 50 Hz with a video camera (Flea3, Point Gray, CA) mounted on a Zeiss Discovery v.12 Stereo Microscope (Carl Zeiss, DE). flyPAD measures fly's behavior on the food source by recording the capacitance at 100 Hz between the electrode on which the fly stands and the food. Videos were compressed to high-quality H.264 encoded files and subsequently manually annotated by a human observer to be used as a benchmark for the development of the automatic algorithms for the extraction of feeding behavior from the capacitive trace. For more info, visit http://flypad.pt/.

### 2AFC task using video triggered reward

Adult PWD female mice were tested in behavioral experiments using restricted social interaction with adult C57BL6 and PWK males as reward. The behavioral paradigm consists of a custom built arena made of modular acrylic pieces assembled in an aluminum frame. The contact zone between the female and the male (composed of four holes with *r* = 0.5 cm) was either available for a fixed period of time, or physically restricted by a vertically sliding door controlled by a servomotor. Subjects initiated the interaction by nose-poking in an infrared beam port that would trigger the opening of the door and subsequent availability of the contact zone. Videos were recorded using high-speed monochrome video cameras (Flea3, Point Gray, CA). Performance, monitoring and control of the behavior box was done using a Motoruino board (Motoruino, Artica, PT) and custom Bonsai scripts.

### Dynamic virtual environment for freely moving rodents

Three to 5 months old Long-Evans female rats were trained sequentially to forage and hunt virtual elements of a projected display in exchange for water rewards. The behavioral paradigm consists of a custom built arena made of structural framing components (Bosch Rexroth, DE). The floor of the arena is a rear-projection screen made out of a frosted acrylic panel. In order to compensate for the short-throw distance, the projected image is reflected off a mirror positioned below the arena floor. Video was recorded using a high-speed monochrome video camera (Flea3, Point Gray, CA) equipped with a visible light cutoff filter (R72, Hoya, JP) and analyzed in real-time using Bonsai. Infrared LED strips were positioned at the bottom of the arena in order to illuminate the floor through the diffuser, allowing for the tracking of the animal without contamination from the visual stimulus. Animals were first conditioned to a tone as a secondary reinforcer and then subsequently trained to either touch the light presented at random locations (foraging) or pursue a moving spot (hunting). Performance, monitoring and control of the behavior box was done using an Arduino board (Micro, Arduino, IT) and a Bonsai reactive state machine.

## Author contributions

Conceived and developed the project: GL, NB, JF; Developed software: GL; Conceived and developed the experiments: GL, NB, JF, JN, BA, SS, LM, SM, PI, PC, RM, LC, ED, JP, AK; Performed and analyzed experiments: GL, JF, JN, BA, SS, LM, SM, PI, PC, RM, LC, ED; wrote the manuscript: GL, AK.

### Conflict of interest statement

The authors declare that the research was conducted in the absence of any commercial or financial relationships that could be construed as a potential conflict of interest.
